# *Vibrio parahemolyticus *septicaemia in a liver transplant patient: a case report

**DOI:** 10.1186/1752-1947-5-171

**Published:** 2011-05-06

**Authors:** Rajeev R Fernando, Sujatha Krishnan, Morgan G Fairweather, Charles D Ericsson

**Affiliations:** 1 Department of Internal Medicine, the University of Texas Health Science Center, 6431 Fannin Street, Houston, Texas 77030, USA

## Abstract

**Introduction:**

*Vibrio parahemolyticus *is the leading cause of vibrio-associated gastroenteritis in the United States of America, usually related to poor food handling; only rarely has it been reported to cause serious infections including sepsis and soft tissue infections. In contrast, *Vibrio vulnificus *is a well-known cause of septicaemia, especially in patients with cirrhosis. We present a patient with *V. parahemolyticus *sepsis who had an orthotic liver transplant in 2007 and was on immunosuppression for chronic rejection. Clinical suspicion driven by patient presentation, travel to Gulf of Mexico and soft tissue infection resulted in early diagnosis and institution of appropriate antibiotic therapy.

**Case presentation:**

A 48 year old Latin American man with a history of chronic kidney disease, orthotic liver transplant in 2007 secondary to alcoholic end stage liver disease on immunosuppressants, and chronic rejection presented to the emergency department with fever, vomiting, abdominal pain, left lower extremity swelling and fluid filled blisters after a fishing trip in the Gulf of Mexico. Samples from the blister and blood grew *V. parahemolyticus*. The patient was successfully treated with ceftriaxone and ciprofloxacin.

**Conclusion:**

Febrile patients with underlying liver disease and/or immunosuppression should be interviewed regarding recent travel to a coastal area and seafood ingestion. If this history is obtained, appropriate empiric antibiotics must be chosen. Patients with liver disease and/or immunosuppresion should be counselled to avoid eating raw or undercooked molluscan shellfish. People can prevent *Vibrio *sepsis and wound infections by proper cooking of seafood and avoiding exposure of open wounds to seawater or raw shellfish products.

## Introduction

*Vibrio parahaemolyticus *is a facultative anaerobic gram-negative, flagellated, halophilic, asporogenous, bacterium that inhabits marine and estuarine environments [1]. Despite its broad distribution, *V. parahemolyticus *infections in the United States of America are most common in individuals living in the states bordering the Gulf of Mexico [2-4]. Water temperature, salinity and turbidity correlate with increased densities of pathogenic *V. parahemolyticus *[2,5]. Filter feeding animals such as shellfish, blue crabs, finfish and planktonic copepods concentrate *V. parahemolyticus*. Consumption of raw or undercooked seafood or exposure of wounds to warm seawater may lead to vibrio infections. The most common clinical presentation is self-limited gastroenteritis (59%), but wound infections (34%), primary septicaemia (5%) and other infection sites (2%) may also occur [3]. Persons who are immunocompromised or who have liver disease are at particularly high risk for severe vibrio infections. Necrotizing soft tissue infections are exceptional and may cause significant morbidity and mortality from invasion and destruction of fascial planes as well as the release of cytokines [6,7].

## Case presentation

**Figure 1:**
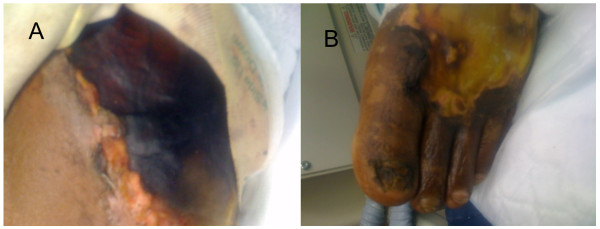
***Vibrio parahemolyticus *cellulitis**. A. Large hemorrhagic bulla of left lower extremity. B. Blistering cellulitis of the left foot. Bedside debridement excluded necrotizing fasciitis.

A 48 year old Latin American male with a history of chronic kidney disease, orthotic liver transplant in 2007 secondary to alcoholic end stage liver disease and chronic rejection on immunosuppressants (tacrolimus, sirolimus, prednisone and mycophenolate mofetil) presented to the emergency department. Two days prior to presentation, patient returned from a fishing trip in the Gulf of Mexico and began to experience fever with chills, vomiting, abdominal pain, left lower extremity pain and swelling. On the evening of his admission he developed multiple clear fluid filled blisters on his left lower extremity extending to his medial thigh (Figure 1); the largest blister measured 6 cm by 7 cm in size. His temperature was 100.2 ˚F; heart rate, 115/min; blood pressure, 92/63 mmHg; and respiratory rate, 34/min. His left lower extremity was warm, tender, erythematous and oedematous with fluid filled blisters and a 2 cm by 2 cm ulcer on the plantar aspect of the left foot. His hemoglobin was 6.6 g/dl with indirect hyperbilirubinemia; LDH, 196 IU/L; white blood cells 6.1 k/ mcl (neutrophils 48%, bands 36%, and lymphocytes 4%), platelets 160 k/ mcl, creatinine 3.1 mg/dl, CK 538 U/L and lactic acid 6.4 mg/ dl. X-rays of the left lower extremity showed diffuse soft tissue swelling without bony involvement. Compression ultrasonography ruled out deep venous thrombosis. A noncontrast computed tomography (CT) of the left lower extremity did not demonstrate muscle necrosis and a CT abdomen showed diffuse colonic thickening. His respiratory status declined and he was intubated. His blood pressure dropped and he was put on two pressors and transfused two units of packed red blood cells and two units of fresh frozen plasma. He was admitted to the critical care unit with a working diagnosis of sepsis and cellulitis. Bedside exploration and subsequent surgical debridement ruled out necrotizing fasciitis. A sample from the blister and blood samples were taken for culture. Stool culture was not performed. Aggressive management with intravenous fluids and empiric treatment with ceftriaxone (2 g intravenously every 24 hours) and ciprofloxacin (400 mg intravenously every 12 hours) was initiated. Blister aspirate and blood cultures were positive for a gram-negative, non-lactose fermenting bacilli. The isolate did not ferment sucrose and yielded round blue-green colonies in thiosulphate citrate bile salt sucrose agar. Microbiologic testing demonstrated *Vibrio parahemolyticus*. Subsequently the patient developed thrombophlebitis and necrotic skin in a circumferential pattern from the ankle to the knee on the left lower extremity that ultimately required debridement and split thickness skin graft. The patient was treated with ceftriaxone and ciprofloxacin and was discharged home in a stable condition.

## Discussion

*Vibrio *species are a rare cause of soft tissue infections. Exceedingly rare is soft tissue infection with *V. parahemolyticus*, which can occur in patients with underlying co-morbidities such as cancer, liver disease, kidney disease, heart disease, recent gastric surgery, or antacid use [4,6]. Wound infection may occur after contamination of skin laceration with warm seawater, after direct trauma with pieces of shellfish, fishhooks or utensils contaminated with seawater or translocation from the gastrointestinal tract [6]. Bacteraemia and septicaemia occur in three to five percent of *Vibrio *infections and is a concern in immunocompromised patients especially those with liver disease [3]. Superficial infection can extend to deeper soft tissue causing cellulitis or necrotizing fasciitis and may require radical surgical debridement [6]. The diagnosis of *V. parahemolyticus *soft tissue infection is difficult. Clinical suspicion must be high in people returning from coastal areas such as the Gulf of Mexico especially with a history of raw seafood consumption or extremity wounds. Soft tissue infections are hard to recognize and difficult to differentiate from necrotizing fasciitis. Our patient underwent a bedside exploration and then debridement to definitively exclude necrotizing fasciitis. *V. parahemolyticus *causes skin and soft tissue necrosis which can further confound the clinical picture. Recognition of necrotizing soft-tissue infections is critical for survival because they may carry a high mortality rate. Surgical debridement must be complemented with broad spectrum antibiotic therapy. *V. parahemolyticus *demonstrates beta-lactamase activity in as many as 50% of isolates [8]. The vibrios are susceptible most notably to fluoroquinolones, third generation cephalosporins and doxycycline, Septicaemia and serious soft tissue infections can be treated with the synergistic combination of ceftazidime plus doxycycline or ceftazidime plus a fluoroquinolone with the latter combination being more potent *in vitro *[9]. There is evidence that patients with cirrhosis and end stage liver disease are susceptible to *Vibrio *infections [10]. This is the first case, however, in which a Vibrio parahemolyticus species infection has been reported in a liver transplant patient. It is imperative to educate patients with compromised liver function of the necessity of avoiding uncooked salt water foods and exposure to brine.

## Conclusion

Preventing contamination of seafood is impossible since several shellfish and finfish filter and concentrate the organism. Raw seafood consumption must be discouraged, particularly for individuals at high risk for development of septicaemia, especially in people with compromised liver function or immunosuppression. Special attention should be paid to possible cross-contamination during the preparation of seafood.

## Competing interests

The authors declare that they have no competing interests.

## Consent

Written informed consent was obtained from the patient for publication of this case report and any accompanying images. A copy of the written consent is available for review by the Editor-in-Chief of this journal.

## Authors' contributions

RF was involved in the management of the patient, performed the literature search and was a contributor in writing the manuscript. MF initiated the preparation of the manuscript and did a literature search. SK was instrumental in preparing the manuscript and performing the literature search. CE helped prepare and edit the manuscript. All authors read and approved the final manuscript.
